# New Insights into the Epigenetics of Hepatocellular Carcinoma

**DOI:** 10.1155/2017/1609575

**Published:** 2017-03-19

**Authors:** Braira Wahid, Amjad Ali, Shazia Rafique, Muhammad Idrees

**Affiliations:** ^1^Centre for Applied Molecular Biology, 87 West Canal Bank Road Thokar Niaz Baig, University of the Punjab, Lahore, Pakistan; ^2^Hazara University, Mansehra, Pakistan

## Abstract

Hepatocellular Carcinoma (HCC) is one of the most predominant malignancies with high fatality rate. This deadly cancer is rising at an alarming rate because it is quite resistant to radio- and chemotherapy. Different epigenetic mechanisms such as histone modifications, DNA methylation, chromatin remodeling, and expression of noncoding RNAs drive the cell proliferation, invasion, metastasis, initiation, progression, and development of HCC. These epigenetic alterations because of potential reversibility open way towards the development of biomarkers and therapeutics. The contribution of these epigenetic changes to HCC development has not been thoroughly explored yet. Further research on HCC epigenetics is necessary to better understand novel molecular-targeted HCC treatment and prevention. This review highlights latest research progress and current updates regarding epigenetics of HCC, biomarker discovery, and future preventive and therapeutic strategies to combat the increasing risk of HCC.

## 1. Introduction

Hepatocellular Carcinoma (HCC) is the fifth most common cancer in the world that causes 250,000 to 1 million deaths annually [1]. This alarming incidence is attributed to several different genetic and epigenetic alterations. HBV and HCV infections, smoking, alcohol, dietary exposure to aflatoxins, diabetes, and obesity are the other risk factors of HCC [2]. Epigenetic changes that contribute to HCC metastasis, invasion, and dissemination encompass noncoding RNAs regulation, DNA methylation, and histone modification. All these changes are associated with initiation and progression of HCC [3].

## 2. Epigenetic Changes in HCC

Epigenetics refers to heritable states of gene expression without alteration to the DNA sequence itself. Epigenetic changes such as DNA hypermethylation or hypomethylation, dysregulation of histone modification patterns, chromatin remodeling, and aberrant expression of micro-RNAs (miRNAs) and long noncoding RNAs (lncRNAs) are associated with HCC [4]. Different epigenetic mechanisms that drive cell proliferation, metastasis, progression, and development of HCC are discussed below.

## 3. DNA Methylation

**Figure 1:**
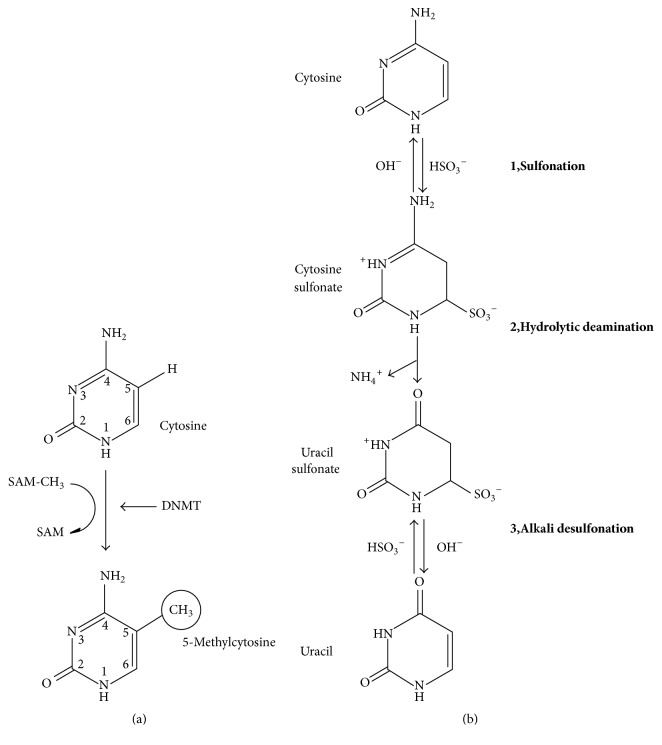
CpG methylation. (a) DNA methylation is catalyzed by three methyl transferase genes (DNMT1, DNMT3a, and DNMT3b) that add methyl group (CH_3_) at 5th carbon position of pyrimidine ring of cytosine. S-adenosyl methionine (SAM-CH3) acts as a methyl donor. (b) Cytosine to cytosine sulfonate: sulfonation of cytosine causes C to T transition followed by deamination. Cytosine sulfonate to uracil sulfonate: conversion of cytosine sulfonate to uracil sulfonate leads to alkali desulfonation. Uracil sulfonate is converted into uracil. PCR distinguishes methylated CpG from unmethylated CpG because methylated cytosine resists this chemical treatment [124, 125].

DNA methylation, specifically methylation of cytosine at 5th carbon, is a well characterized epigenetic mechanism of gene regulation that occurs in mammals at promoter-rich region of gene that is, cytosine-phosphate-guanine (CpG) (Figure 1).

CpG dinucleotides occur throughout human genome in nonuniform manner with the frequency of about one per eighty nucleotides [5]. Approximately 1 to 2% of human genome is referred to as CpG islands or CpG-rich regions containing hundred to several thousand base pairs and exists in proximity to different gene promoter regions [6]. Nearly 70% of human genes harbor CpG islands at 5′ region that consist of promoter as well as transcription sites [7].

In HCC and other wide range of tumors, specific promoter hypermethylation and global hypomethylation have been associated with inactivation of tumor-suppressor genes (TSGs) and genomic instability, respectively. Silencing of tumor-related genes and tumor-suppressor genes such as* SOCS1*,* hMLH1*, and* RASSF1A* is achieved by hypermethylation of CpG islands in promoter sequences that downregulates mRNA transcript expression. Epigenetic silenced genes play an important role in molecular pathways of carcinogenesis such as cell adhesion or DNA repair, apoptosis, and cell cycle regulation [8]. Set of proteins known as ten-eleven translocation (Tet1–3) demethylates methylcytosine via hydroxymethylcytosine (hmC) [9]. It has been reported that level of hmC reduces in various types of cancers [10]. However, the mechanism of this downregulation is still to be determined [11].

Different DNA methylome patterns as compared to adjacent normal tissues have been observed in independent genome-wide methylation profiling studies. HCC and non-HCC surrounding liver tissues can be distinguished easily because aberrant DNA hypermethylation is specific to the cancerous tissues. Likewise, a set of hypermethylated gene promoters, for example,* FZD7*,* CDKN2A*,* RASSFIA*, and* APC*, were able to distinguish nontumor liver tissues from HCC tumors. Another study recruited 27 patients and revealed the significant level of DNA methylation of* NFATC1*,* GSTP1*,* CDKN2A*, and* BMP4* genes in HCC tissues [12]. Lambert et al. have recently analyzed the methylation status of set of imprinted genes in HCC and found that 15q11-13 imprinting control region that includes maternally imprinted* GABRA5* gene was significantly hypomethylated in tumors compared to their surrounding tissues. The study suggested that imprinted gene methylation acts as a potential marker of environmental exposures [13].

Poor tumor differentiation is attributed to promoter methylation of* DNMT1* [14, 15].* S100A8* can be used as prognostic and diagnostic biomarker because of its overexpression observed in MHCC-97H and Huh-7 cell lines [16]. Likewise, HK2 promoter CpG island (HK2-CGI) represents prognostic biomarker of HCC because hypermethylation of HK2-CGI induces HK2-CGI methylation phenotype (HK2-CIMP) [17] (Table 1).

Other frequently methylated genes in HCC include* RIZ1* (45.2%),* CDKN2A* (69.7%),* SCARA5* (30%),* EFEMP1* (50%),* TIP30* (47%),* WIF1*,* FBLN1* (50%),* DLEC1* (70.6%),* FBP1* (80%),* ITGA4* (23%),* KLK10* (94%),* LIFR* (47.9%),* MTIG* (60.4%),* HHIP* (53.6%),* HINT1* (55%),* SYK* (12%), and* TAT* (54%) [18, 19].

Bead array analysis of 1505 CpG sites in thirty HBV- or HCV-associated HCC infected patients revealed the correlation of specific methylation signatures with tumor progression stage in HCC tumor patients. Hypermethylation of* SYK* (spleen tyrosine kinase) or* CHFR* (checkpoint with fork-head associated and ring finger) occurs specifically in advanced stages of HCC, whereas abnormal DNA methylation of* p15*,* GAAD45a*,* SFRP1*,* DOK1*,* CHRNA3*,* GSTP1*,* CRABP1*,* p16*, and* RASSF1A* occurs at all stages of HCC [20–23]. HBV-encoded protein (HBx) affects the methylation and expression by directly interacting with* DNMT* (DNA-methyltransferase). The prevalence of TSGs methylation, for example,* p15*,* APC*,* STAT1*,* GADD45b*, and* SOCS-1*, has been observed to be higher in HCV-positive HCC compared to HCV-negative HCC [24, 25]. Hernandez-Vargas et al. revealed that panel of hypermethylated genes, for example,* DCC*,* CSPG2*, and* NAT2,* was specific to HBV-related HCC [26, 27] and methylation of constitutive androstane receptor (CAR) suppresses* CYP2C19* in HBV-associated HCC patients [28]. According to Shih et al. methylation of* PAX6* frequently occurs in HCV-associated HCC tissues (61.3%) compared to HBV positive (22.1%) and double negative HCC tissues (33.3%) [29].

A genome-wide methylation study including 69% HBV-associated HCC patients demonstrated that* PAX4*,* WFDC6*,* SCGB1D1*,* ATK3*, and* CCL20* were top five hypomethylated genes, whereas* CDKN2A*,* SPDY1*,* ZFP41*,* BMP4*, and* DAB2IP* were found to be top five hypermethylated genes [30]. Nishida et al. demonstrated that DNA methylation is an important mechanism in silencing the set of 8 TSGs that predict HCV progression to HCC [31].* NEFH* and* SMPD3* have been proved as potent tumor-suppressor genes in HCC [32]. Based on the above findings it can be concluded that early diagnosis and prognosis of HCC can be achieved by methylation profiling because of close association of aberrant gene methylation with clinical outcome and HCC disease stage. However, specific gene methylation signatures must be validated [26]. Recent study has suggested that DNA methylation of miRNA genes may provide a promising strategy for alternative adjuvant therapy in HCC [33].

## 4. Histone Modification

Histone modifications also known as “histone code” have direct impact on gene expression and chromatin structure. These epigenetic changes are of paramount importance in gene silencing during tumorigenesis [107]. Modifications such as ubiquitination, phosphorylation, methylation, and acetylation that occur at N-terminal tails of nucleosomal histones work together with other epigenetic mechanisms to regulate gene activities and cellular processes. Orderly and coordinated activities of diverse histone modifications regulate cellular processes, for example, DNA repair, DNA replication, and gene transcription. It has been reported that control of chromatin-based processes (responsible for cancer development and oncogenic transformation) is deregulated by functional changes in protein complexes and histone-modifying molecules [108].

DNA methylation is closely associated with histone modification because inhibitors reverse the histone modification changes on H3-K4 and H3-K9 codes [109, 110]. Another distinct histone modification involved in Polycomb-based silencing and X-chromosome inactivation in women is H3K27 trimethylation that is one of the candidates for a silencing mechanism for tumor-suppressor genes [111]. Overexpression of enhancer of zeste homolog 2 (EZH2) is associated with different types of cancer and it has been studied that EZH2 catalyzes histone H3-K27 triMe [112]. Histone methyltransferases (HMT), SUV39H1 and G9a, mediate the histone H3-K9 trimethylation and dimethylation (H3-K9 diMe), respectively [113]. H3K9 methylation is associated with silencing of several tumor-suppressor genes [114]. Significant decrease in histone H2A ubiquitination was noticed in HCC [115].

Researchers have reported several histone modifications that are associated with HCC and alter normal cellular processes; for example, Magerl et al. found negligible expression of dimethylation of histone H3 at lysine 4 (H3K4diMe) in HCC [116] whereas, according to Shon et al., Patt1 (a GNAT family acetyltransferase) is downregulated in HCC and it is overexpressed in healthy liver [117]. Somatic mutation that induced inactivation of MLL1–5 enzymes responsible for H3KYMe has been reported in 1–6% of HCC [118]. Integration of HBV into* MLL2* and* MLL4* gene loci has also been identified in many cases of HCC [119]. In contrast to this, elevated H3K4me3 is associated with mutations in SMYD3 methyltransferase resulting in poor prognosis specifically during the initial stages of HCC [120]. Likewise, upregulation of SETDB1, another histone methyltransferase for H3K9, was observed in HCC. Overexpression of SETDB1 that is closely associated with metastasis and cancer progression actually is triggered by downregulation of miR-29 and gain of chromosome 1q21 [121, 122]. About 2.6% of cancer patients experience mutations in SETD2 gene [118, 123].

Histone deacetylases (HDACs) are the enzymes that play an important role in regulation of gene expression by removing acetyl group from histones that make the DNA more compact leading to gene silencing. About 18 HDACs are known with activity not limited to just histones because it has been reported that HDACs remove acetyl-lysine on diverse nonhistone proteins like NFkB, transcription factors p53, and many others [128].

Accumulating evidence suggests the correlation of individual HDACs overexpression with poor prognosis in different types of cancer including HCC [129]. Overexpression of HDAC3 was correlated with early recurrence of HCC after surgery and advance tumor stage. Tumor-suppressor role of HDACs has also been noticed as overt HCC that occurred as a result of liver-specific knockdown of HDAC3 [130]. Specifically, persistent inactivation of SMRT, NCOR, or HDAC3 may lead to cancer development and DNA damage by increasing the histone acetylation during S phase [131]. HDACs may act as tumor suppressors and therapeutic targets in developing tumors and advanced cancer, respectively [132]. Inhibition of HDAC may disturb a drug design due to disruption of diverse pathways.

Some histone modifications also act as signature for risk factor exposures; for example, protein arginine methyltransferase 1 (PRMT1) catalyzes histone H4 methylation on arginine 3 and dephosphorylates damage-induced phosphorylation of H2AX (g-H2AX) to repair DNA. In case of HCV infection, Protein Phosphatase 2A (PP2Ac) is overly expressed and inhibits the activity of PRMT1 by binding to it. Overexpression of this phosphatase is considered an important event of viral hepatitis associated hepatocarcinogenesis. Compromised histone H2AX phosphorylation and histone H4 acetylation/methylation occur because of PP2Ac overexpression in HCV-associated HCCs leading to significant changes in gene expression for hepatocarcinogenesis and inhibition of DNA damage repair. Overexpression of this phosphatase is considered a critical early event in hepatocarcinogenesis in the context of chronic viral hepatitis [24, 133].

The direct interaction of HBx with histone acetyltransferase complex CBP/P300 supports the transcription transactivation property of an oncogenic transcription factor HBV-encoded HBx protein that affects the expression of different genes involved in apoptosis or cell cycle control. Recruitment of CBP/P300 complex that is mediated by HBx promotes transactivation and leads to acetylated (active) state of the target cellular genes [117, 134, 135]. Downregulation of* CYP2E1* expression in response to deregulated histone modification which resulted in decline in apoptotic potential has been observed in alcohol-associated HCCs [136, 137]. Recent studies suggest that HCC progression can be repressed by inhibition of O-GlcNAcylation [138]. Epigenetic silencing of JMJD5 (jumonji C domain-containing protein 5), another tumor-suppressor gene in HCC pathogenesis, downregulates* CDKN1A* transcription to promote HCC cell proliferation [139].

## 5. Chromatin Remodeling

**Figure 2:**
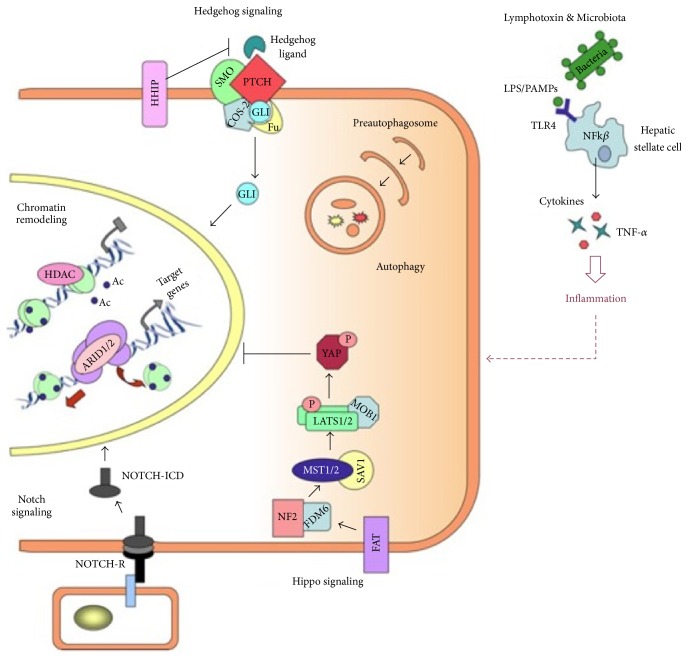
Emerging signaling pathways in HCC: chromatin remodeling: restricting transcriptional and DNA condensation occurs as a result of histone deacetylation catalyzed by HDACs in nucleosome. In contrast, transcriptional activation also occurs using chromatin remodeling complexes by allowing access to transcription machinery via nucleosome restructuring. Notch signaling: NOTCH receptor is cleaved photolytically when protein ligand binds to its extracellular domain. This binding releases its intracellular domain (NOTCH-ICD) that enters into nucleus to modify target gene expression (such as* SOX9*,* HEY*, and* HES*). Hedgehog (Hh) signaling: nuclear translocation of the transcription factor (TF) GLI occurs as a result of PTCH inhibitory effect on SMO and this event takes place in the presence of Hh signaling. Hippo signaling: kinase complexes Lats1/2-Mob1 and MST1/2-SVA1 are activated with phosphorylation of the transcription factor YAP resulting in prevention of its nuclear translocation. This event involves the use of upstream regulators of hippo pathway (i.e., FDM6, NF2, and FAT). Microbiota and lymphotoxins: NF-k*β* signaling activates and produces proinflammatory molecules such as TNF-*α* and cytokines due to recognition of microbial ligands (LPS/PAMPs) by TLKRs (e.g., TLK4) on the hepatic stellate cells [126].

Another important epigenetic mechanism that plays an important role in control of gene expression, differentiation, DNA repair, and proliferation is chromatin remodeling (Figure 2).

Nucleosomal restructuring by ATP-dependent chromatin remodeling complexes and enzymatic covalent histone modifications are the principal mechanisms involved in chromatin remodeling. Accumulating evidence in recent years has demonstrated that these complexes perform tumor-suppressor roles because of association of different malignancies with inactivated mutations [140]. Recent study based on SWI/SNF chromatin remodeling complex revealed that expression of brahma (BRM) was markedly decreased in HCC samples [141].* ARID1A*,* ARID1B*, and* ARID2* components that belong to SWI/SNF-related chromatin remodeling complexes are mutated at frequency of 16.8%, 6.7%, and 5.6%, respectively [142].* ARID1A*,* ARID1B*, and* ARID2* mutations are significantly observed in alcohol-associated HCC and HCV-related HCC, respectively [143]. Mutations in other components of SWI/SNF chromatin remodeling complex such as* SMARCC2*,* SMARCC1*,* SMARCB1*,* SMARCA4*, and* SMARCA2* have also been commonly reported in HCCs.* SMARCA2* mutations occur at frequency of 2.6% in alcohol-associated HCC [118]. Chromatin remodeler* CHD1L* that promotes HCC metastasis and progression may act as therapeutic target to control HCC [144]. The analysis of whole exome sequencing of 24 HCCs revealed that chromatin regulators are the third most frequently mutated genes [142].

## 6. Noncoding RNAs

**Figure 3:**
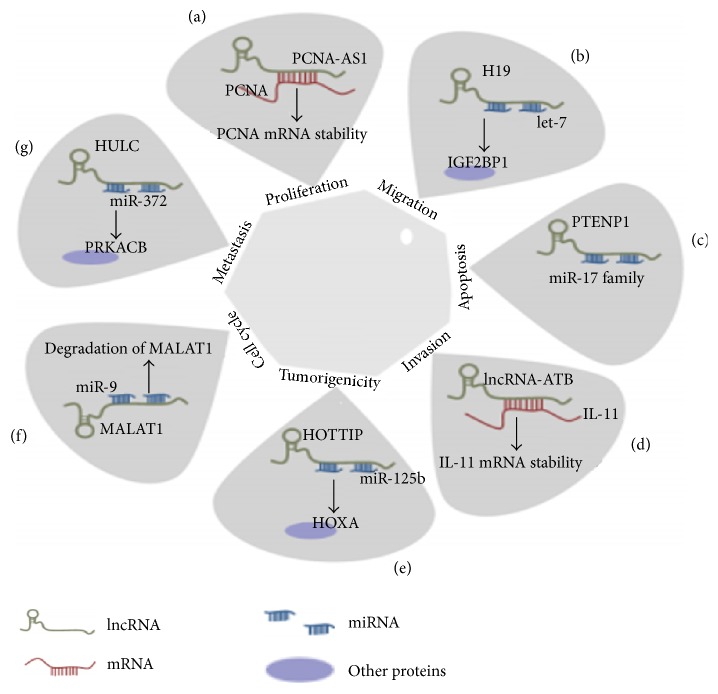
Role of miRNA, mRNA, and lncRNA in regulation of apoptosis, migration, metastasis, tumorigenicity, cell cycle, invasion, and cell proliferation. PCNA (Proliferating Cell Nuclear Antigen) regulation is related to PCNA-AS1 effects and this event involves the formation of RNA hybridization that increases PCNA mRNA stability. (b) H19 affects let-7 mediated genes involved in promotion of metastasis specifically IGF2BP1 (insulin-like growth factor 2 mRNA-binding protein). (c) Autophagy genes, for example, p62, ATG7 (autophagy-related gene 7), and ULK1 (unccoordinated-51- (unc-51-) like kinase 1), are regulated by PTENP1 that is targeted by miR-17 family. (d) The stability of IL-11 (interleukin-11) mRNA is increased by lncRNA-ATB. (e) miR-125b negatively targeted by HOTTIP. (f) The regulation of MALAT1 is regulated in the nucleus after being binded with miR-9 following AGO2-dependent path. (g) The activity and expression of miR-372 are repressed after getting binded with HULC. Target mRNA of miR-372, that is, Prkacb (cAMP-dependent protein kinase catalytic subunit beta), level is increased in response to mi-372 reduction [127].

Different human cancers are associated with noncoding RNAs based epigenetic mechanism of regulation of gene expression (Figure 3).

Noncoding RNAs are further categorized into two main types based on length: small/short noncoding RNAs that are <200 nucleotides including endogenous siRNAs, snoRNAs, piRNAs, and miRNAs and long noncoding RNAs with length >200 nucleotides [145, 146]. Different functions of miRNA are described in Table 2.

Significant decrease in miRNA-129-2 with associated inhibition of HMGB1 (high mobility group box 1) has been observed in HCC [51]. miRNA122 that regulates Wnt1, igF1R, SRF, ADAM10, and cyclin G1 to play an active role in cell cycle progression is downregulated in HCC [44]. miRNA-125b and miRNA-26 induce cell-cycle arrest in HCC by targeting oncogenic LIN28B and cyclin D2/cyclin E2, respectively [43, 50]. Overexpression of miRNA-96 is observed in HBV-related HCC. In HCC cell lines, miRNA-101 targets VEGF-C resulting in the suppression of invasion [147]. miRNA-21 has been found upregulated in HCC and its degradation can be used as target in therapeutics [148]. Likewise, recent study has demonstrated the tumor-suppressor activity of miRNA-214 by inhibiting CDK6, CDK3, and E2F2 [149]. In addition to inhibition of cell proliferation of HCC by miRNA-449a, lentivirus mediated overexpression of miRNA-199a, miRNA-133b, and miRNA-185 has also been reported [53, 59, 150, 151]. Oncogenic miRNA-221 and miRNA-1180 target cell cycle inhibitors (CDKN1C/p57, CDKN1B/p27) and repress TNIP2 expression, respectively, resulting in increased proliferation of HCC cells [39, 152]. Likewise, cell proliferation is suppressed in HCC due to let-7a and let-7 g that regulates the oncogenic STAT3 and cMyc, respectively [42]. miRNA-135a targets fork-head box O1 and miRNA-155-3p suppresses FBXW7 leading to an increased invasion and migration in HCC [56, 153]. miRNA-186 targets Yes-associated protein 1 and acts as a potential therapeutic target in treating HCC [48].

Long noncoding RNAs also play an important role in different types of cancers including HCC. Scaffold, signal, guide, and decoy are the molecular mechanisms of lncRNAs [154, 155]. The most widely studied lncRNAs are described in Table 3.

Potential utilization of noncoding RNAs as novel candidates in treatment, detection, diagnosis, and prognosis of HCC are promising. During the last decade, accumulating evidence suggests the use of noncoding RNAs as potential therapeutic targets for HCC. However, pathological and biological aspects as well as molecular mechanism of noncoding RNAs in HCC are an emerging area of science that needs more research to develop potential therapeutic intervention and treatment against HCC.

## 7. Epigenetic Biomarkers of HCC

Epigenetic alterations such as DNA hypermethylation, DNA hypomethylation, and noncoding RNAs or histone modifications may serve as diagnostic and prognostic biomarkers of HCC.

Overexpression of histone phosphorylation proteins such as* ARK1* and* ARK2* and histone-modifying genes, such as histone methyltransferases* G9a*,* EZH2*, and* SUV39HZ*, in HCC tissues predicts tissue invasion and poor prognosis [130, 156].

DNA methylation acts as potential biomarker of HCC because of higher frequency of aberrant methylation found in HCC tissues that can help a clinician or researcher to distinguish healthy liver from cirrhotic liver or liver of HCC patient [157]. Likewise, deregulated expression level of several noncoding RNAs can be used for diagnosis and prognosis of disease. Downregulation of miR-122 is associated with poor prognosis of HCC [158]. Studies showed decreased expression of miR199a/b-3pis and increased expression of miR-21 in HCC tissues [159]. Deregulation of long noncoding RNA has also been reported in HCC tissues. Upregulation of HOTTIP and HOXAIR in HCC tissues has been associated with poor patient survival, tumor progression, and metastasis [74]. Recently, researchers predicted HCC with 100% specificity and 95.6% sensitivity based on DNA methylation level approach in preneoplastic liver tissue [160].

All these findings are encouraging to develop an epigenetic-based biomarker; however, more research regarding specificity, sensitivity, and reproducibility is needed to make the usability of methylated DNA, modified histones, and noncoding RNAs as novel and reliable biomarkers.

## 8. Nutritional Epigenetics

Occurrence of HCC can be reduced and the development of HCC can be delayed because of epigenetic mechanisms deregulated by nonnutrient dietary bioactive components and several different nutrients. Liver carcinogenesis is under the influence of chemopreventive potential of epigenetic food components such as dietary methyl-group donors, that is, sulforaphane, sodium butyrate, curcumin, resveratrol, and epigallocatechin-3-gallate (ECGC). Food-based deregulation of epigenome contributes towards HCC-related angiogenesis, oxidative stress, apoptosis, inflammation, and cell proliferation.

Several in vivo and in vitro preclinical models of HCC have demonstrated the antihepatocarcinogenic effects of polyphenolic compound curcumin that demethylates DNA and expresses DNMTs to reactivate abnormally silenced cancer related genes [161]. Green tea beverages are enriched with EGCG that have enough potential to inhibit progression and development of HCC via DNA demethylation of abnormally hypermethylated tumor-suppressor genes [162]. Two coffee polyphenols, that is, chlorogenic acid and caffeic acid, inhibited liver carcinogenesis in rat and the inhibition of human DNMT1-mediated enzymatic DNA methylation reaction has also been reported [163, 164].

Abundant HDAC inhibitors are of natural origin and present in different plants such as broccoli (sulforaphane), grapes (resveratrol), blueberries (piceatannol), and garlic (allyl mercaptan) [165]. Bioactive polyphenol resveratrol produced naturally in blueberries, grapes, and strawberries exhibited chemopreventive effects in liver cancer by promoting apoptosis [166]. In addition to this, resveratrol induced anticancer properties such as inflammation and attenuation of oxidative stress in hepatocarcinogenesis [167, 168].

Sodium butyrate produced as a result of metabolic degradation of carbohydrates in human colon and an isothiocyanate sulforaphane found in cruciferous vegetables prevent HCC by inhibiting HDACs [169, 170]. In a nutshell, nutritive epigenetics may serve as potential strategy to prevent HCC progression.

## 9. Epigenetic-Based Therapeutics for HCC and Future Updates

It is evident that epigenetic alterations play an important role in HCC and therefore can be targeted for treatment. In fact, in recent years the epigenetic drugs are in progress that reverse histone modification and methylation status. Combination of epigenetic drugs may also treat HCC (Table 4).

HHC patients can be treated with HDAC inhibitors such as valproic acid, TSA, panobinostat, ITF2357, resminostat, givinostat, abexinostat, CUDC-101, and pracinostat that have given encouraging results in HCC patients because aberrant expression of HDAC is higher in cancer patients [101, 106, 171–174]. Epigenetic drugs can stimulate the immunity of host by increasing tumor antigen presentation [175].

MiRNAs with tumor-suppressor nature are perfect anticancer agents because of their ability to modulate multiple signaling pathways in cancer growth. Modulation of expression of miRNAs or targeting any tumor-related deregulated ncRNA may offer potential new therapeutic strategies.

Histone-modifying enzymes and DNMTs are the prime candidates for future HCC therapy. Series of clinical studies are currently under research for the development of epigenetic-based therapies to combat life-threatening condition HCC.

## 10. Conclusion

The above studies provide strong evidence that epigenetic alterations are in close association with disease stage and clinical outcome in HCC. The best-known genetic abnormalities in HCC are dysregulated expression of epigenetic regulatory genes, aberrant expression of noncoding RNAs, promoter methylation, and DNA methylation. Several different epidrugs target these aberrations and control the progression of HCC by reversing the expression of cell cycle and apoptosis related genes. Epigenetic alterations can potentially be considered an alternative option in cancer treatment protocols because epigenetic changes are reversible unlike genetic changes that are irreversible. Epigenetic changes affect cellular transcriptome alterations and result in gene expression and chromatin organization more extensively than genetic changes. Many of drugs with the potential to change the pattern or level of histone modification and DNA methylation have been developed and are now in clinical trials. As prognostic and diagnostic biomarker of HCC, it has been shown that histone modification, DNA methylation, and differential expression of noncoding RNAs help researchers to distinguish between HCC and cirrhotic liver or between tumor and nontumor adjacent tissues. Multikinase inhibitor, Sorafenib, is the first drug that treats HCC. HCC can be suppressed by inhibition of expression or activity of key proteins involved in carcinogenesis; therefore, epigenetic modulation of histones and the expression regulation of miRNAs serve as useful therapeutic strategies against HCC. Detailed mechanisms of HCC-related epigenetic-based therapeutics remain to be explored. Last but not least, targeted and efficient use of epigenetic drugs makes them prime candidates for future HCC therapy.

## Figures and Tables

**Table 1:** Aberrant DNA methylation markers for HCC.

Gene and its location	Function	Methylation frequency% in adjacent normal tissue	Methylation frequency% in HCC	Ref.
*WT1*	Urogenital development	0	54	[34]
*11p13*				
*TIMP3*	Cell adhesion	0	13	[35]
22q12.3				
*SOCS-1*	Cytokine inhibitor	0–7	43–65	[36]
16p13.13				
*SEMA3B*	Apoptosis	—	83	[37]
3p21.3				
*RB*	Chromatin structure	0	32	[34]
13q14.2				
*RASSF1A*	Apoptosis	0	59–75	[35, 37]
3p21.3				
*RaR-Beta*	Retinoic acid signaling	7	12	[36]
3p24.2				
*P73*	Tumor suppressor	0	6	[36]
1p36.32				
*P53*	Tumor suppressor	0	14	[34]
17p13.1				
*P300*	Growth/cell division	0	65	[34]
22q13.2				
*P27*	CDK inhibitor	0	48	[34]
12p13.1				
*P21*	CDK inhibitor	10	63	[34]
6p21.2				
*P16* ^*INK4a*^	CDK inhibitor	0–10	16–83	[35, 36]
9q21.3				
*P15*	CDK inhibitor	0	42–47	[36]
18q12.2				
*P14*	CDK inhibitor	0	6	[36]
11q13.1				
*hMLH1*	Mismatch repair	0	0	[35]
3p21.3				
*GSTP1*	Glutathione synthesis	0–7	41–76	[35, 36]
11q13				
*E-Cadherin*	Cell adhesion	7	33–67	[34–36]
16q22.1				
*E2F-1*	Transcription factor	0	70	[34]
20q11.22				
*DAPK1*	Apoptosis	0	10	[35]
9q21.33				
*CPS1*	Urea cycling	0	80	[38]
2q346				
*COX2*	Prostaglandin synthesis	0	35–50	[35]
1q31.1				
*BLU*	Unknown zinc-finger	—	20	[37]
3q21.3				
*APC*	Prostaglandin synthesis	0–14	53–81	[35, 36]
5q22.2				

**Table 2:** Role of different miRNAs in HCC.

miRNA	Function of miRNA	Gene target involved	Reference
miRNA-221	Oncogenic	DDIT4, CDKN1C/p57, CDKN1B/p27	[39]
miRNA-125b	Tumor suppressor	LIN28B	[40]
miRNA-214	Tumor suppressor	c-Myc, TCF-1, Cyclin D1	[41]
let-7 family	Tumor suppressor	c-Myc, STAT3	[42]
miRNA-26	Tumor suppressor	MMP2, cyclin D1, Mcl-1, Bcl-2	[43]
miRNA-122	Tumor suppressor	ADAM10, igF1R, SRF, Cyclin G1, Wnt1, AKT3, Bcl-w	[44, 45]
miRNA-96	Oncogenic	ephrinA5	[46]
miRNA-101	Tumor suppressor	Mcl-1	[47]
miRNA-186	Tumor suppressor	Yes-associated protein 1	[48]
miRNA-29	Tumor suppressor	Mcl-2, Bcl-2	[49]
miRNA-125b	Tumor suppressor	ILL- 6R, Bcl-w, Mcl-1, Bcl-2	[50]
miRNA-129-2	Tumor suppressor	High mobility group box 1	[51]
miRNA-193a-3p	Oncogenic	SRSF2	[52]
miRNA-133b	Tumor suppressor	SIRT1	[53]
miRNA-199a-3p	Tumor suppressor	c-Met, mTOR	[54]
miRNA-199a-5p	Tumor suppressor	ATG7	[55]
miRNA-155-3p	Oncogenic	FBXW7	[56]
miRNA-222	Oncogenic	PPP2R2A, p27	[57]
miRNA-21	Oncogenic	PTEN, kinase 3	[58]
miRNA-449a	Tumor suppressor	ADAM10	[59]
miRNA-139	Tumor suppressor	ROCK2, Rho Kinase 2	[60]
miRNA-125b	Tumor suppressor	LIN28B, PDZ binding motif, Sirtuin 7	[40, 50, 60]
miRNA-182	Oncogenic	ephrinA5	[46]
miRNA-125a	Tumor suppressor	VEGF-A, MMP11	[61]
miRNA-1180	Increase apoptotic resistance to HCC	Through activation of NF-*κ*B pathway	[62]
miRNA-200 family	Tumor suppressor	ZEB2, ZEB1	[63]
miRNA-212	Tumor suppressor	FOXA1	[64, 65]
miRNA-497	Tumor suppressor	YAP1	[66]
miRNA-519d	Metastasis	PTEN	[67]
miRNA-106b	Apoptosis	Bim	[68]

**Table 3:** The potential roles of widely studied HCC-related lncRNAs.

lncRNA	Potential role in HCC	Ref.
HOTAIR	Overexpression of HOTAIR is associated with progression of HCC via activation of Wnt/*β*-catenin signaling pathway. HOTAIR upregulates expression of ATG3 and ATG7 that ultimately activate autophagy and promote HCC cell proliferation.	[69, 70]
CCAT1	CCAT1 functions as let-7 sponge and increases HCC progression.	[71]
HULC	HULC increases the metastasis and tumorigenesis of HCC through miR-200a-3p/ZEB1 signaling pathway.	[72]
H19	Low expression of H19 decreases HCC progression and metastasis via upregulation of miR-200 family.	[73]
HOTTIP	HOTTIP overexpression is associated with metastasis in HCC patients. This lncRNA is negatively regulated by miR-125b.	[74, 75]
BANCR	BANCR is considered as an important contributor of progression and initiation of HCC and therefore can be used as biomarker.	[76]
MALAT1	MALAT1 is associated with tumor progression because of its upregulation in HCC cell lines.	[77]
HEIH	HEIH is oncogenic in nature and promotes tumor progression.	[78]
PTENP1	PTENP1 represses tumorigenic properties of HCC cells.	[79]
SNHG20	SNHG20 is upregulated in HCC and may serve as prognostic biomarker of HCC.	[80]
MEG3	Tumor suppressor MEG3 is associated with pathogenesis of HCC malignancy.	[81]
TUC338	Upregulation of TUC338 and TUC339 modulates cell growth and increases liver cirrhosis.	[82, 83]
LINC-ROR	LINC-ROR acts as mediator of chemotherapeutic response and increases chemosensitivity in HCC because HCC is highly resistant to chemotherapy. It also promotes cell survival during hypoxia.	[84, 85]
MVIH	MVIH confirms overall-survival and recurrence-free survival.	[86]
lncRNA-ATB	lncRNA-ATB acts as a mediator of TGF-*β* signaling that increases metastasis in HCC.	[87]
TUG1	Upregulation of TUG1 in HCC and increasing apoptosis and cell growth by epigenetic silencing of KLF2.	[88]
URHC	URHC expression is increased in HCC tissues. It regulates apoptosis and cell proliferation via downregulation of ZAK.	[89]
FTX	Binds to miR-374a and MCM2 and inhibits metastasis and proliferation in HCC.	[90]
PVT1	High expression level of PVT1 is linked with tumor progression and may act as biomarker of tumor recurrence in HCC patients.	[91]
lncRNA-p21	lncRNA-p21 is downregulated in HCC and its overexpression inhibits tumor invasion by inhibiting Notch signaling and EMT.	[92]
UCA1	Upregulation of UCA1 is associated with progression of HCC via activation of FGFR1-ERK signaling pathway and inhibition of miR-216b.	[93]
MT1DP	MT1DP acts as tumor suppressor and inhibits FOXA1 in liver cancer cells because of negative regulation of MT1DP by YAP and RUNx2.	[94]
UFC1	Upregulation of HFC1 promotes cell cycle progression and HCC cell proliferation.	[95]
SRHC	Downregulation of SRHC inhibits cancer proliferation; however, the epigenetically silenced SRHC promotes proliferation in HCC.	[96]
PCNA-AS1	PCNA-AS1 can serve as therapeutic target because it promotes tumor growth in HCC.	[97]
lncRNA-LET	Downregulation of LET is associated with reduction in HCC metastasis and invasion.	[79]
lncRNA-Dreh	lncRNA-Dreh is important in tumor suppression. Downregulation of Dreh inhibits HCC metastasis by targeting vimentin.	[98]
UCA1/CUDR	UCA1/CUDR is involved in chemotherapeutic resistance.	[99]

**Table 4:** Drugs that target epigenetic modifications in HCC [3].

Epigenetic modification	Drugs	Results	Ref.
Histone deacetylation that targets histone deacetylase	Suberoylanilide hydroxamic acid	TNF-related apoptosis-inducing ligand-induced apoptosis.	[100]
	Belinostat	Tumor stabilization was observed in nonresectable advanced HCC.	[101]
	Belinostat	Induction of apoptosis and inhibition of cell growth occurred.	[102]

DNA methylation that targets DNA methyltransferases	5-Aza-2′-deoxycytidine	Inhibition of telomerase activity and reactivation of c-Myc and p16 were observed.	[103]
	Zebularine	Induction of apoptosis and inhibition of cell proliferation were observed in HepG2 cell line.	[104]
	Zebularine	Tumor growth was inhibited in xenograft models. Genes involved in apoptosis, cell cycle, and tumor suppression were demethylated in KMHC and Huh-7 cell lines.	[105]

Combined epigenetic modifications that target tyrosine kinase inhibitors and histone deacetylase inhibitors	Panobinostat + sorafenib	Combined activity of these two drugs induced apoptosis, increased survival, and decreased tumor density and tumor volume.	[106]
